# Associations of body mass index and sarcopenia with screen-detected mild cognitive impairment in older adults in Colombia

**DOI:** 10.3389/fnut.2022.1011967

**Published:** 2022-10-18

**Authors:** Gary O’Donovan, Olga L. Sarmiento, Philipp Hessel, Graciela Muniz-Terrera, Claudia Duran-Aniotz, Agustín Ibáñez

**Affiliations:** ^1^Latin American Brain Health Institute (BrainLat), Universidad Adolfo Ibáñez, Santiago, Chile; ^2^Facultad de Medicina, Universidad de los Andes, Bogotá, Colombia; ^3^Swiss Tropical and Public Health Institute, Basel, Switzerland; ^4^Heritage College of Osteopathic Medicine, Ohio University, Athens, OH, United States; ^5^Centre for Social and Cognitive Neuroscience, School of Psychology, Universidad Adolfo Ibáñez, Santiago, Chile; ^6^Global Brain Health Institute, University of California, San Francisco, San Francisco, CA, United States; ^7^Trinity College Dublin, Dublin, Ireland; ^8^Centro de Neurociencias Cognitivas, Universidad de San Andrés, Buenos Aires, Argentina; ^9^Trinity College Institute of Neuroscience (TCIN), Trinity College Dublin, Dublin, Ireland

**Keywords:** cognition, overweight, obesity, sarcopenia, South America

## Abstract

**Background and objective:**

More research is required to understand associations of body mass index (BMI) and sarcopenia with cognition, especially in Latin America. The objective of this study was to investigate associations of BMI and sarcopenia with mild cognitive impairment in Colombia.

**Design, setting, and participants:**

Data were from the National Survey of Health, Wellbeing and Aging in Colombia (SABE Colombia, in Spanish). Community-dwelling adults aged 60 years or older were invited to participate.

**Methods:**

Trained interviewers administered a shorter version of the mini-mental state examination and mild cognitive impairment was defined as a score of 12 or less out of 19. Body mass index was defined using standard cut-offs. Sarcopenia was defined as low grip strength or slow chair stands. Logistic regression models were adjusted for age, sex, height, education, income, civil status, smoking, and alcohol drinking.

**Results:**

The prevalence of mild cognitive impairment was 20% in 23,694 participants in SABE Colombia and 17% in 5,760 participants in the sub-sample in which sarcopenia was assessed. Overweight and obesity were associated with decreased risk of mild cognitive impairment and sarcopenia was associated with increased risk. Sarcopenia was a risk factor for mild cognitive impairment in those with normal BMI (adjusted model included 4,911 men and women). Compared with those with normal BMI and without sarcopenia, the odds ratio for mild cognitive impairment was 1.84 in those with normal BMI and sarcopenia (95% confidence interval: 1.25, 2.71). Sarcopenia was also a risk factor in those with obesity but did not present a greater risk than sarcopenia alone. Compared with those with normal BMI and without sarcopenia, the odds ratio was 1.62 in those with obesity and sarcopenia (95% confidence interval: 1.07, 2.48). Sarcopenia was not a risk factor for mild cognitive impairment in those with overweight. Similar results were observed when reference values from Colombia were used to set cut-offs for grip strength. Similar results were also observed in cross-validation models, which suggests the results are robust.

**Conclusion:**

This is the first study of the combined associations of sarcopenia and obesity with cognition in Colombia. The results suggest that sarcopenia is the major predictor of screen-detected mild cognitive impairment in older adults, not overweight or obesity.

## Introduction

The proportion of people living with dementia is predicted to increase by around 75% by 2050 in the UK and other countries in western Europe and by around 200% in Colombia and other countries in Latin America (1). It is important to identify modifiable risk factors because a 5-year delay in onset might halve the prevalence of dementia (2). Nearly all the evidence about potentially modifiable risk factors for cognitive impairment comes from studies in high-income countries and there is an urgent need for more evidence from low-income and middle-income countries, particularly from Latin America (3). A risk factor is an exposure or a behavior that is causally related to a health problem and the existence of causal evidence in high-income countries does not negate the need for causal evidence elsewhere (4, 5). Indeed, consistency of association is one of the fundamental considerations when inferring causality (4, 5). Sarcopenia is a muscle disease characterized by low muscle strength (6) and evidence from high-income countries suggests that sarcopenia is a risk factor for cognitive impairment (7). Obesity is a risk factor for cognitive impairment (3) and evidence from high-income countries also suggests that sarcopenic obesity presents a greater risk for cognitive impairment than sarcopenia alone or obesity alone (8, 9). We are not aware of any studies of the combined associations of sarcopenia and obesity with cognitive impairment in Colombia. There are several mechanisms that may explain why sarcopenic obesity presents a greater risk for cognitive impairment (10). For example, sarcopenia may cause an imbalance in the secretion of myokines and memory impairment (11) and sarcopenic obesity may exacerbate the secretion of pro-inflammatory myokines (10).

In modern epidemiology, it is understood that cross-sectional studies can provide important information about etiology (12). For example, the difference in prevalence between two exposure groups can be viewed as a causal effect itself (12). It is also understood that it is important to gather evidence from each country in Latin America because the region is so difficult to comprehend (13). There is great cultural and ethnic diversity in Latin America (13). There are also varying levels of income and healthcare in the region (13, 14). Colombia may be particularly difficult to understand because the country has suffered 50 years of civil war (15). The National Survey of Health, Wellbeing and Aging in Colombia (SABE Colombia, according to its initials in Spanish) is the largest nationally representative survey of older adults in Colombia (16). The aim of the present analysis was to investigate associations of obesity and sarcopenia with mild cognitive impairment in SABE Colombia.

## Materials and methods

### Participants

The National Survey of Health, Wellbeing and Aging in Colombia is described in detail elsewhere (16). Briefly, the target population was community-dwelling adults aged 60 years or older living in urban and rural areas of Colombia. Participants were selected using a multistage area probability sampling design and there were four selection stages: municipalities, blocks, housing units, and households. The response rate was around 62% in urban areas, around 77% in rural areas, and around 70% overall (16). Data were collected across all departments (that is, states) and the final sample was deemed to be representative of the population of older adults living in households in Colombia (16). Sarcopenia was assessed in a sub-sample that was also designed to be nationally representative (17). People living in 86 of 244 municipalities in Colombia were invited to participate, including people living in the four large cities of Bogotá, Medellín, Cali, and Barranquilla (17). The sample size in the sub-sample was 6,161, assuming that the proportion of older adults was 6%, that the maximum error was 6%, and that the non-response rate was 20% (17). The final sub-sample included 5,760 people, which is equivalent to 93% of the target sample size. Trained interviewers conducted face-to-face interviews in the participant’s home between April and September 2015 (16). Volunteers completed the shorter version of the Folstein Mini-Mental State Examination (MMSE) described below and were invited to complete the survey if they had a score of 13 or more (16). Otherwise, a friend or family member was invited to complete the survey on behalf of the participant. A friend or family member completed the interview in 17.5% of cases (16). Institutional review boards of Universidad de Caldas (CBCS-021-14) and Universidad del Valle (09–014 and 011–015) approved the study and all participants gave written informed consent.

### Outcome variable

The outcome variable was mild cognitive impairment. The versions of the MMSE used in Latin America and the Caribbean are shorter than the original version in an attempt to reduce the low literacy bias (18). The shorter version of the MMSE used in SABE Colombia has six questions and participants were asked: to state the date and the day of week (4 points); to repeat and remember three words (3 points); to state in reverse order the numbers 1, 3, 5, 7, 9 (5 points); to take a piece of paper in their right hand, fold it in half using both hands, and put it on their lap (3 points); to reiterate the three words given earlier (3 points); and, to copy a drawing of two overlapping circles (1 point). A score of 12 or less out of 19 was used to screen for mild cognitive impairment in SABE Colombia (16). The shorter version of the MMSE used in SABE Colombia has been validated in a study of 1,301 adults aged 60 years or older living in households in Chile (19). The prevalence of mild cognitive impairment was 10.7% using the threshold of 12 or less out of 19 in the shorter version of the MMSE and 8.1% using the threshold of 6 or more out of 33 in the criterion measure (19), which was the Short Portable Mental Status Questionnaire (20).

### Exposure variables

The main exposure variables were BMI and sarcopenia. Participants were asked to take off their shoes and to remove any heavy objects, such as coins, keys, and coats. Trained interviewers measured weight and height, and body mass index was expressed as weight in kilograms divided by height in meters squared. Body mass index values greater than 75 were deemed to be dubious and were not included in the present analysis. Body mass index cut-offs were defined using World Health Organization guidelines: normal was 18.5–24.9 kg/m^2^; underweight was < 18.5 kg/m^2^; overweight was 25.0–29.9 kg/m^2^; and, obesity was ≥ 30 kg/m^2^ (21). Analyses were adjusted for height because of the inverse association between BMI and height in older adults (22).

Sarcopenia, or muscle failure, is a muscle disease and the European Working Group on Sarcopenia in Older People 2 (EWGSOP2) uses low muscle strength as the primary parameter of sarcopenia because muscle strength is presently the most reliable measure of muscle function (6). The EWGSOP2 guidelines recommend that grip strength and chair stands be used to assess muscle strength (6). Grip strength was assessed using the Smedley III dynamometer (Takei Scientific Instruments, Tokyo, Japan). The interviewer adjusted the dynamometer so that it fitted the participant’s hand. The interviewer also explained how to use the dynamometer and gave a demonstration. The participant was instructed to stand with their feet hip-width apart, to hold their arm to the side without touching their body, and to squeeze the device as hard as possible. Grip strength was assessed twice in each hand with 30 s of rest between tests. The highest value was used in the present analysis. Low grip strength was defined using the EWGSOP2 cut-offs of < 27 kg in men and < 16 kg in women (6). Participants who could not complete the assessment were included in the low grip strength group. Chair stands were assessed using a sit-to-stand test. The interviewer explained how to perform the test and gave a demonstration. The participant was instructed to stand up as quickly as possible five times without stopping. The participant was asked to keep their arms crossed against the chest and was told that they would be timed. A slow sit-to-stand test was defined using the EWGSOP2 cut-off of > 15 s in men and women (6). Participants who could not complete the assessment were included in the slow chair stands group. The EWGSOP2 recommends that muscle strength be assessed to screen for sarcopenia and that muscle quality be assessed to confirm sarcopenia (grip strength and chair stands can be used to asses muscle strength and magnetic resonance imaging, computed tomography, dual-energy X-ray absorptiometry, and bioelectrical impedance can be used to assess muscle quality) (6). The present study was designed to screen for “probable sarcopenia” and was not designed to confirm the diagnosis (16, 17).

### Covariates

The analyses were adjusted for a range of covariates that may be associated with cognition, including age, sex, education, income, civil status, cigarette smoking, and alcohol drinking (23, 24). Participants were asked about the highest level of education they had achieved, and three groups were created: no education; some primary education; and, some secondary education or more. Participants were also asked about their current individual income according to multiples of the minimum wage. Participants were asked about their current civil status, and two groups were created: not married or with partner; and, married or with partner. Participants were asked about cigarette smoking, and three groups were created: never, former smoker, and current smoker. Participants were also asked about alcohol drinking in the last month, and two groups were created: non-drinker; and, drinker. The trained interviewers also measured height.

### Sensitivity analyses

The cut-offs used in the main analysis were based on European populations and may not be applicable to Colombia (6). Grip strength was assessed in 672 men and 487 women aged 18–64 years who took part in a nationally representative survey in Colombia between 2015 and 2016 (25, 26). Peak grip strength values were 38.9 ± 10.2 in men and 23.6 ± 5.3 in women (mean ± standard deviation) (25). In sensitivity analyses, cut-offs were set two standard deviations below these reference values for grip strength for adults in Colombia: < 18.5 kg in men and < 13 kg in women. We are not aware of any reference values for chair speed for adults in Colombia.

### Statistical analysis

Analyses were performed using Stata SE version 17.0 for Mac (StataCorp, Texas, USA). Logistic regression was used to investigate associations of BMI and sarcopenia with mild cognitive impairment. Logistic regression models were adjusted for age and sex and further adjusted for height, education, current individual income, civil status, cigarette smoking, and alcohol drinking. Age and height were modeled as continuous variables. All other covariates were modeled as categorical variables. Odds ratios and 95% confidence intervals were calculated for all participants. Cross-fit, partialing-out lasso logistic regression was used to test the robustness of the models. In lasso regression, the dataset can be split and cross-validated. The coefficients are obtained from one sample and used in another, which is independent, and that adds robustness. Here, the dataset was split ten times and the “controls” were automatically selected from the covariates chosen *a priori* (i.e., age, sex, height, education, current individual income, civil status, cigarette smoking, and alcohol drinking). The output includes odds ratios and the Wald statistic. Odds ratios are interpreted in the same way as in ordinary logistic regression models. The Wald statistic indicates whether the exposure is statistically significant.

## Results

Table 1 shows the characteristics of the 23,694 participants in SABE Colombia and the 5,760 participants in the sub-sample in which sarcopenia was assessed. The sample and the sub-sample were similar. For example, age was 71 ± 8 years in the sample and 71 ± 8 years the sub-sample. The proportion of males was 43% in the sample and 40% in the sub-sample. The prevalence of mild cognitive impairment was 20% in the sample and 17% in the sub-sample. The proportion of normal weight was 31% in the sample and 30% in the sub-sample. The proportion of obesity was 19% in the sample and 20% in the sub-sample. The prevalence of sarcopenia was 44% when defined as low grip strength and 58% when defined as slow chair stands. Supplementary Figure 1 shows the flow of data in the present analysis.

**TABLE 1:** Characteristics of participants in the National Survey of Health, Wellbeing and Aging in Colombia and the sub-sample in which sarcopenia was assessed.

	SABE Colombia	
	Survey	Sub-sample
**Age in years, mean ± SD (n)**	71 ± 8 (23,694)	71 ± 8 (5,760)
**Sex**		
Male, *n* (%)	10,112 (42.68)	2,325 (40.36)
Female, *n* (%)	13,582 (57.32)	3,435 (59.64)
Missing, *n* (%)	0 (0)	0 (0)
Total, *n* (%)	23,694 (100)	5,760 (100)
**Mild cognitive impairment**		
No, *n* (%)	19,004 (80.21)	4,789 (83.14)
Yes, *n* (%)	4,690 (19.79)	971 (16.86)
Missing, *n* (%)	0 (0)	0 (0)
Total, *n* (%)	23,694 (100)	5,760 (100)
**MMSE score, mean ± SD (*n*)**	14.94 ± 3.91 (23,694)	15.24 ± 3.77 (5,760)
**Body mass index**		
Normal, *n* (%)	7,347 (31.01)	1,710 (29.69)
Underweight, *n* (%)	771 (3.25)	160 (2.78)
Overweight, *n* (%)	7,678 (32.40)	1,991 (34.57)
Obesity, *n* (%)	4,452 (18.79)	1,161 (20.16)
Missing, *n* (%)	3,446 (14.54)	738 (12.81)
Total, *n* (%)	23,694 (100.00)	5,760 (100)
**Grip strength**		
Normal, *n* (%)	–	3,212 (55.76)
Low, *n* (%)	–	2,548 (44.24)
Missing, *n* (%)	–	0 (0)
Total, *n* (%)	–	5,760 (100)
**Chair stands**		
Normal, *n* (%)	–	2,401 (41.68)
Slow, *n* (%)	–	3,359 (58.32)
Missing, *n* (%)	–	0 (0)
Total, *n* (%)	–	5,760 (100)
**Height in cm, mean ± SD (*n*)**	156 ± 9 (20,585)	156 ± 9 (5,114)
**Education**		
None, *n* (%)	5,229 (22.07)	1,035 (17.97)
Some primary, *n* (%)	13,462 (56.82)	3,399 (59.01)
Some secondary or more, *n* (%)	4,910 (20.72)	1,299 (22.55)
Missing, *n* (%)	93 (0.39)	27 (0.47)
Total, *n* (%)	23,694 (100.00)	5,760 (100)
**Civil status**		
Not married or with partner, *n* (%)	11,127 (46.96)	2,699 (46.86)
Married or with partner, *n* (%)	12,557 (53.00)	3,061 (53.14)
Missing or did not answer, *n* (%)	10 (0.04)	0 (0)
Total, *n* (%)	23,694 (100.00)	5,760 (100)
**Current income**		
None, *n* (%)	3,736 (15.77)	823 (14.29)
Less than minimum wage, *n* (%)	13,468 (56.84)	3,129 (54.32)
Minimum wage, *n* (%)	3,168 (13.37)	882 (15.31)
1–2 Times minimum, *n* (%)	1,906 (8.04)	547 (9.50)
2–3 Times minimum, *n* (%)	570 (2.41)	162 (2.81)
3–4 Times minimum, *n* (%)	262 (1.11)	58 (1.01)
More than 4 times minimum, *n* (%)	229 (0.97)	54 (0.94)
Missing or did not answer, *n* (%)	355 (1.50)	105 (1.82)
Total, *n* (%)	23,694 (100.00)	5,760 (100)
**Cigarette smoking**		
Never smoked, *n* (%)	11,400 (48.11)	2,885 (50.09)
Former smoker, *n* (%)	9,763 (41.20)	2,291 (39.77)
Current smoker, *n* (%)	2,523 (10.65)	580 (10.07)
Missing or did not answer, *n* (%)	8 (0.03)	4 (0.07)
Total, *n* (%)	23,694 (100.00)	5,760 (100)
**Alcohol drinking**		
Non-drinker, *n* (%)	20,881 (88.13)	5,076 (88.12)
Drinker, *n* (%)	2,796 (11.80)	681 (11.82)
Missing or did not answer, *n* (%)	17 (0.07)	3 (0.05)
Total, *n* (%)	23,694 (100.00)	5,760 (100.00)

Data are from the National Survey of Health, Wellbeing and Aging in Colombia (SABE Colombia). Participants were community-dwelling adults aged 60 years or older. Mild cognitive impairment was defined as a score of 12 or less out of 19 on the shorter version of the mini-mental state examination (MMSE) used in SABE Colombia, which is a valid screening tool. Body mass index was defined using World Health Organization categories, where normal is 18.5–24.9 kg/m^2^, underweight is < 18.5 kg/m^2^, overweight is 25.0–29.9 kg/m^2^, and obesity is ≥ 30 kg/m^2^. The cut-off for low grip strength was < 27 kg in men and < 16 kg in women. The cut-off for slow chair stands was > 15 s in men and women. N is number and SD is standard deviation.

Body mass index was assessed in 20,248 participants. Table 2 shows associations between BMI and mild cognitive impairment. Compared with those with normal BMI, the odds ratio for mild cognitive impairment was 1.63 in those with underweight after adjusting for age and sex (95% confidence interval: 1.37, 1.95). Compared with those with normal BMI, the odds ratio for mild cognitive impairment was 0.68 in those with overweight (95% confidence interval: 0.62, 0.75) and 0.63 in those with obesity (95% confidence interval: 0.56, 0.71). Similar associations were observed after further adjustment for height, education, income, civil status, smoking, and alcohol drinking.

**TABLE 2:** Associations of body mass index with mild cognitive impairment.

	Body mass index			
	Normal	Underweight	Overweight	Obesity
**Model 1**	1.00 (reference) *n* = 7,347 (21% with MCI)	1.64 (1.37, 1.95) *n* = 771 (33% with MCI)	0.68 (0.62, 0.75) *n* = 7,678 (13% with MCI)	0.63 (0.56, 0.71) *n* = 4,452 (12% with MCI)
**Model 2**	1.00 (reference) *n* = 7,191 (20% with MCI)	1.61 (1.34, 1.95) *n* = 752 (33% with MCI)	0.76 (0.69, 0.84) *n* = 7,532 (13% with MCI)	0.74 (0.65, 0.83) *n* = 4,385 (12% with MCI)

Values are odds ratios (95% confidence intervals). Mild cognitive impairment (MCI) was defined as a score of 12 or less out of 19 on the shorter version of the mini-mental state examination used in SABE Colombia, which is a valid screening tool. Model 1 was adjusted for age and sex (n = 20,248). Model 2 was adjusted for age, sex, height, education, income, civil status, smoking, and alcohol drinking (n = 19,860).

Grip strength and chair stands were assessed in 5,760 participants. Table 3 shows associations between grip strength and mild cognitive impairment. Compared with those with normal grip strength, the odds ratio for mild cognitive impairment was 2.00 in those with low grip strength after adjusting for age and sex (95% confidence interval: 1.70, 2.35). Similar associations were observed after further adjustment for height, education, income, civil status, smoking, and alcohol drinking. Table 3 also shows associations between chair stands and mild cognitive impairment. Compared with those with normal chair stands, the odds ratio for mild cognitive impairment was 1.66 in those with slow chair stands after adjusting for age and sex (95% confidence interval: 1.39, 1.97). Similar associations were observed after further adjustment for height, education, income, civil status, smoking, and alcohol drinking.

**TABLE 3:** Associations of grip strength and chair stands with mild cognitive impairment.

	Normal	Low or slow
***Grip strength***		
Model 1	1.00 (reference)	2.00 (1.70, 2.35)
Model 2	1.00 (reference)	1.90 (1.56, 2.31)
***Chair stands***		
Model 1	1.00 (reference)	1.66 (1.39, 1.97)
Model 2	1.00 (reference)	1.61 (1.31, 1.98)

Values are odds ratios (95% confidence intervals). Mild cognitive impairment was defined as a score of 12 or less out of 19 on the shorter version of the mini-mental state examination used in SABE Colombia, which is a valid screening tool. The cut-off for low grip strength was < 27 kg in men and < 16 kg in women. The cut-off for slow chair stands was > 15 s in men and women. Model 1 was adjusted for age and sex (n = 5,760 for grip strength and chair stands, including 17% with mild cognitive impairment). Model 2 was adjusted for age, sex, height, education, income, civil status, smoking, and alcohol drinking (n = 5,000 for grip strength and chair stands, including 14% with mild cognitive impairment).

Table 4 shows combined associations of BMI and sarcopenia with mild cognitive impairment when sarcopenia is defined as either low grip strength or slow stair stands. The results suggest that sarcopenia is a risk factor for mild cognitive impairment in those with normal BMI. For example, compared with those with normal BMI and without sarcopenia, the odds ratio for mild cognitive impairment was 1.84 in those with normal BMI and sarcopenia after adjusting for age, sex, height, education, income, civil status, smoking, and alcohol drinking (95% confidence interval: 1.25, 2.71). The results also suggest that sarcopenia is a risk factor in those with obesity. For example, compared with those with normal BMI and without sarcopenia, the odds ratio was 1.62 in those with obesity and sarcopenia after adjusting for all the covariates (95% confidence interval: 1.07, 2.48). Conversely, the results suggest that sarcopenia is not a risk factor for mild cognitive impairment in those with overweight. For example, compared with those with normal BMI and without sarcopenia, the odds ratio was 1.23 in those with overweight and sarcopenia after adjusting for all the covariates (95% confidence interval: 0.83, 1.83). The effect of underweight was unclear because of the low number of participants with the condition. Combined associations of body mass index and sarcopenia with mild cognitive impairment were similar when sarcopenia was defined as low grip strength alone (Supplementary Table 1) and when sarcopenia was defined as slow chair stands alone (Supplementary Table 2). However, the association of sarcopenic obesity with mild cognitive impairment was not statistically significant when sarcopenia was defined as slow chair stands (odds ratio: 1.33; 95% confidence interval: 0.93, 1.91).

**TABLE 4:** Combined associations of body mass index and sarcopenia with mild cognitive impairment, where sarcopenia is defined as low grip strength or slow chair stands.

	Body mass index			
	Normal	Underweight	Overweight	Obesity
***Model 1***				
No sarcopenia	1.00 (reference) *n* = 547 (8% with MCI)	2.02 (0.66, 6.22) *n* = 30 (17% with MCI)	0.51 (0.31, 0.84) *n* = 671 (4% with MCI)	0.86 (0.51, 1.45) *n* = 400 (6% with MCI)
Sarcopenia	1.82 (1.27, 2.60) *n* = 1,163 (22% with MCI)	1.62 (0.94, 2.81) *n* = 130 (25% with MCI)	1.21 (0.84, 1.73) *n* = 1,320 (15% with MCI)	1.42 (0.96, 2.09) *n* = 761 (15% with MCI)
***Model 2***				
No sarcopenia	1.00 (reference) *n* = 537 (8% with MCI)	1.81 (0.57, 5.80) *n* = 28 (28% with MCI)	0.58 (0.34, 0.99) *n* = 660 (4% with MCI)	1.01 (0.58, 1.74) *n* = 399 (6% with MCI)
Sarcopenia	1.84 (1.25, 2.71) *n* = 1,134 (22% with MCI)	1.50 (0.82, 2.74) *n* = 125 (24% with MCI)	1.23 (0.83, 1.83) *n* = 1,283 (14% with MCI)	1.62 (1.07, 2.48) *n* = 745 (15% with MCI)

Values are odds ratios (95% confidence intervals). Mild cognitive impairment (MCI) was defined as a score of 12 or less out of 19 on the shorter version of the mini-mental state examination used in SABE Colombia, which is a valid screening tool. Sarcopenia was defined as low grip strength (< 27 kg in men and < 16 kg in women) or slow chair stands (> 15 s in men and women). Model 1 was adjusted for age and sex (n = 5,022). Model 2 was adjusted for age, sex, height, education, income, civil status, smoking, and alcohol drinking (n = 4,911). N is number.

The reference values for adults aged 18–64 years in Colombia were used to set the cut-offs for grip strength in the sensitivity analyses. The prevalence of low grip strength was 67% when the country-specific cut-offs were used. Not only was the prevalence of low grip strength higher, but the association between low grip strength and mild cognitive impairment was stronger when the country-specific cut-offs were used. Compared with those with normal grip strength, the odds ratio for mild cognitive impairment was 2.13 in those with low grip strength after adjusting for age, sex, height, education, income, civil status, smoking, and alcohol drinking (95% confidence interval: 1.53, 2.98). Table 5 shows combined associations of BMI and sarcopenia with mild cognitive impairment using the country-specific cut-offs for grip strength. Sarcopenia was a risk factor for mild cognitive impairment in those with normal weight and in those with obesity in the main analysis and in the sensitivity analysis. Sarcopenia was not a risk factor in those with overweight in the main analysis or the sensitivity analysis.

**TABLE 5:** Combined associations of body mass index and sarcopenia with mild cognitive impairment, where sarcopenia is defined using low grip strength reference values from Colombia.

	Body mass index			
	Normal	Underweight	Overweight	Obesity
***Model 1***				
No sarcopenia	1.00 (reference) *n* = 723 (12% with MCI)	1.96 (0.85, 4.53) *n* = 46 (20% with MCI)	0.62 (0.43, 0.90) *n* = 722 (7% with MCI)	0.82 (0.48, 1.40) *n* = 249 (8% with MCI)
Sarcopenia	2.15 (1.48, 3.11) *n* = 987 (22% with MCI)	1.76 (0.98, 3.12) *n* = 114 (25% with MCI)	1.39 (0.95, 2.05) *n* = 1,269 (13% with MCI)	1.69 (1.12, 2.56) *n* = 912 (13% with MCI)
***Model 2***				
No sarcopenia	1.00 (reference) *n* = 708 (12% with MCI)	1.64 (0.67, 4.02) *n* = 46 (20% with MCI)	0.72 (0.48, 1.09) *n* = 708 (7% with MCI)	1.04 (0.58, 1.85) *n* = 247 (7% with MCI)
Sarcopenia	2.25 (1.51, 3.35) *n* = 963 (22% with MCI)	1.70 (0.90, 3.22) *n* = 107 (24% with MCI)	1.45 (0.95, 2.21) *n* = 1,235 (13% with MCI)	1.96 (1.25, 3.08) *n* = 897 (13% with MCI)

Values are odds ratios (95% confidence intervals). Mild cognitive impairment (MCI) was defined as a score of 12 or less out of 19 on the shorter version of the mini-mental state examination used in SABE Colombia, which is a valid screening tool. Sarcopenia was defined using grip strength reference values from a nationally representative survey of 1,159 adults aged 18–64 years in Colombia: < 18.5 kg in men and < 13 kg in women. Model 1 was adjusted for age and sex (n = 5,022). Model 2 was adjusted for age, sex, height, education, income, civil status, smoking, and alcohol drinking (n = 4,911). N is number.

Supplementary Table 3 shows associations of BMI with mild cognitive impairment in the original model and the cross-validation model. Supplementary Table 4 shows associations of grip strength and chair stands with mild cognitive impairment in the original models and the cross-validation models. Supplementary Figure 2 shows combined associations of BMI and sarcopenia with mild cognitive impairment in the original model and the cross-validation model. The odds ratios and the 95% confidence intervals were similar in the original models and the cross-validation models and the exposures were highly significant, which suggests that the models are robust and that sound inferences can be made.

## Discussion

The aim of the present analysis was to investigate associations of BMI and sarcopenia with mild cognitive impairment in older adults in Colombia. We found that overweight and obesity were associated with decreased risk of mild cognitive impairment. We also found that sarcopenia was associated with increased risk of mild cognitive impairment, whether defined as low grip strength or slow chair stands. Sarcopenic obesity did not present a greater risk for mild cognitive impairment than sarcopenia alone. Similar results were observed when reference values from Colombia were used to set the cut-offs for grip strength. Similar results were also observed in the cross-validation models, which suggests that the results are robust. Cross-sectional studies are an important first step in epidemiology and this study could pave the way for longitudinal studies in Colombia.

It is plausible that sarcopenia is associated with increased risk of cognitive impairment. Physical exercise increases the production of brain-derived neurotrophic factor and other myokines associated with brain metabolism and memory (27). Physical exercise also improves brain vascular function and cognition (28). Conversely, sarcopenia is associated with an imbalance in the secretion of myokines and memory impairment (11). Sarcopenia is also associated with vascular dysfunction and cognitive impairment (11). It is also plausible that sarcopenic obesity presents a greater risk for cognitive impairment (10). In sarcopenic obesity, lipids accumulate in skeletal muscle and induce mitochondrial dysfunction and enhanced secretion of pro-inflammatory myokines (10). There is no consensus on the definition of sarcopenic obesity (6) and it is not clear whether sarcopenia or obesity drives the pathology of the condition (10).

Relatively small cross-sectional studies of some 350 older adults in New York in the US (9) and some 1,600 older adults in Tokyo in Japan (8) suggest that sarcopenic obesity presents a greater risk for cognitive impairment. Our relatively large cross-sectional study is in agreement with a recent longitudinal study in showing that sarcopenia is the major predictor of cognitive function in older adults, not obesity (29). Batsis et al. followed 5,822 older adults in the US for 8 years (29). Compared with those without sarcopenia or obesity, the risk of impaired cognitive function was no different in obesity alone (hazard ratio: 0.98; 95% confidence interval: 0.82, 1.16), but was significantly higher in sarcopenia (hazard ratio: 1.60; 95% confidence interval: 1.42, 1.80) and sarcopenic obesity (hazard ratio: 1.20; 95% confidence interval: 1.03, 1.40) (29). Longitudinal studies in Europe and North America also suggest that overweight and obesity are risk factors for dementia in midlife, but not in later life (30, 31). These longitudinal studies in Europe and North America suggest that it is the trajectory of change rather than the current BMI that is most useful in identifying those who are more likely to develop mild cognitive impairment or dementia (31). Indeed, BMI may fall around 6–10 years before diagnosis (31). We are not aware of any such longitudinal studies in Colombia or elsewhere in Latin America.

Debates about cut-off points have hampered research in the field of sarcopenia because of a lack of study consistency (6). The EWGSOP2 uses low muscle strength as the primary parameter of sarcopenia and recommends cut-offs for grip strength and chair stands to increase the harmonization of sarcopenia studies (6). The EWGSOP2 acknowledges that its recommendations focus on European populations and suggests that local reference values be used to set cut-offs when available (6). The EWGSOP2 cut-offs and local reference values were used in the present study. The prevalence of sarcopenia was higher when reference values for Colombian adults were used to define low grip strength; however, any reclassification did not affect associations of BMI and sarcopenia with mild cognitive impairment. The Colombian Ministry of Sport provides free exercise classes to older adults in various towns and cities throughout the country (32) and it would have important implications for policymakers and members of the public if sarcopenia were a stronger predictor of cognitive impairment than obesity. Weight loss is difficult to achieve (33), but physical exercise and a healthy diet may slow or reverse sarcopenia in older adults (34, 35).

This study has some strengths and limitations. There is an urgent need for more evidence from Latin America about modifiable risk factors for cognitive impairment (3). The main strength of the present analysis is that we used data from the largest nationally representative study of older adults in Colombia to investigate associations of BMI and sarcopenia with mild cognitive impairment. It is noteworthy that we used EWGSOP2 cut-offs and local reference values to define sarcopenia. It is also noteworthy that we used cross-validation to test the robustness of the models. The main limitation is the cross-sectional design. Being underweight was associated with increased risk of mild cognitive impairment in the present study; however, there were few too participants with underweight to allow us to fully understand associations of underweight and sarcopenia with mild cognitive impairment. Any association between underweight and cognitive impairment might be explained by reverse causality, where brain pathology causes weight loss prior to the diagnosis of cognitive impairment or dementia (23). Cohort studies with long follow-up times are needed to clarify associations of BMI and sarcopenia with cognitive impairment. Some variables were self-reported and are subject to biases. The shorter version of the MMSE used in SABE Colombia is a valid screening tool (19), but it is not a clinical diagnosis of cognitive impairment. The assessment of muscle strength is a valid screening tool, but it is not a clinical diagnosis of sarcopenia (6). The prevalence of sarcopenia was higher when defined as slow chair stands than when defined as low grip strength in the present study and more research is required to determine which test of muscle strength is more strongly associated with mild cognitive impairment. The sample may have been healthier than the sub-sample and associations of BMI and sarcopenia with mild cognitive impairment may have been underestimated. Participants in the present study lived through more than 50 years of civil war and future generations may have different risk factor profiles with the implementation of the peace deal. For example, future generations may benefit from higher levels of education and may suffer from higher levels of obesity.

## Conclusion

Nearly all the evidence about potentially modifiable risk factors for cognitive impairment has come from high-income countries and more evidence from low-income and middle-income countries has been called for (3). The National Survey of Health, Wellbeing and Aging in Colombia is the largest nationally representative study of older adults in Colombia. The present analysis suggests that sarcopenia is the major predictor of mild cognitive impairment in older adults in Colombia, not overweight or obesity. Sarcopenia is avoidable and more should be done to encourage physically active lifestyles and healthy diets in Colombia because the proportion of people living with dementia is predicted to increase dramatically by 2050 (1).

## Data availability statement

The data analyzed in this study is subject to the following licenses/restrictions: The user agreement does not permit sharing the data directly. Requests to access these datasets should be directed to the Colombian Ministry of Health (repositorio@minsalud.gov.co). The original name of the study is in Spanish: Salud, Bienestar, and Envejecimiento (SABE Colombia).

## Ethics statement

The studies involving human participants were reviewed and approved by the Universidad de Caldas, Colombia and Universidad del Valle, Colombia. The patients/participants provided their written informed consent to participate in this study.

## Author contributions

GO’D: conceptualization, methodology, validation, formal analysis, and writing—original draft preparation. OS, PH, GM-T, CD-A, and AI: writing—reviewing and editing. All authors contributed to the article and approved the submitted version.
