# Supplementary material for: Associations of body mass index and sarcopenia with screen-detected mild cognitive impairment in older adults in Colombia
Source: Front Nutr. 2022 Oct 18;9:1011967. doi: 10.3389/fnut.2022.1011967 (PMC9623159; doi:10.3389/fnut.2022.1011967)
Supplement: Supplementary file 1 [file Data_Sheet_1.docx]

**Associations of body mass index and sarcopenia with mild cognitive impairment in older adults in Colombia**


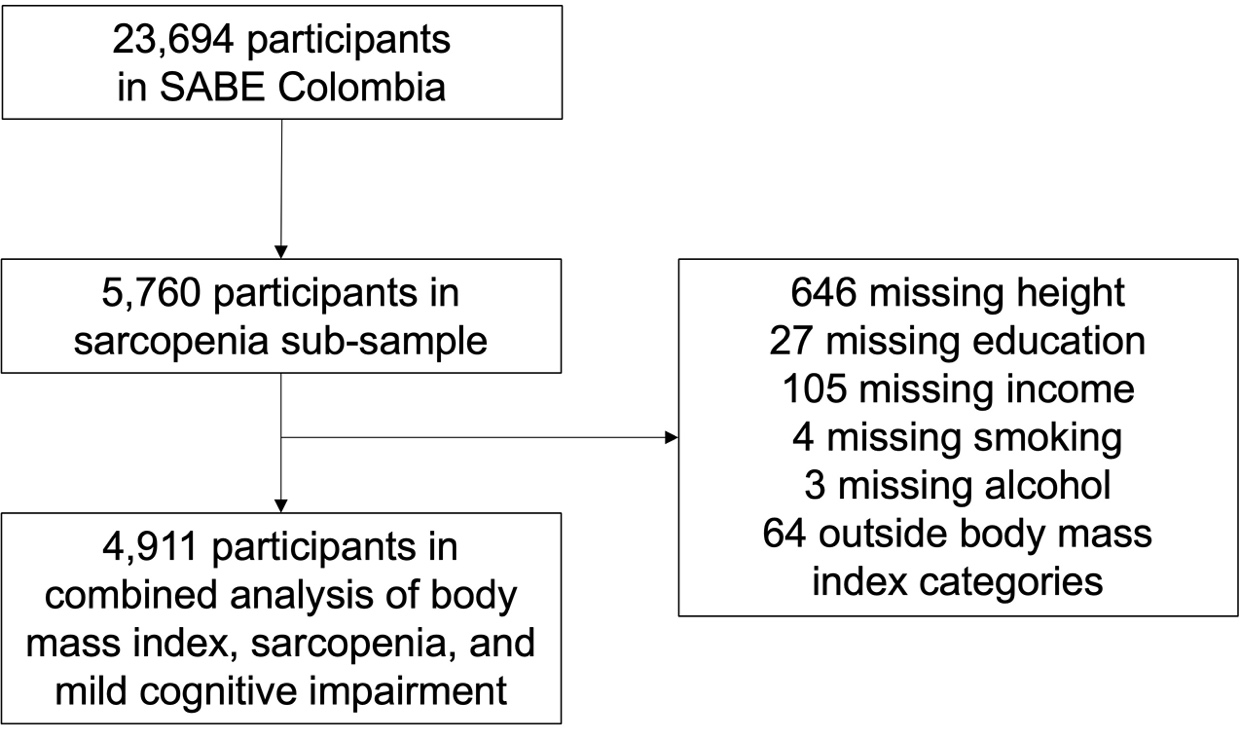


**Supplementary Figure 1.** Flow of data in the present analysis.


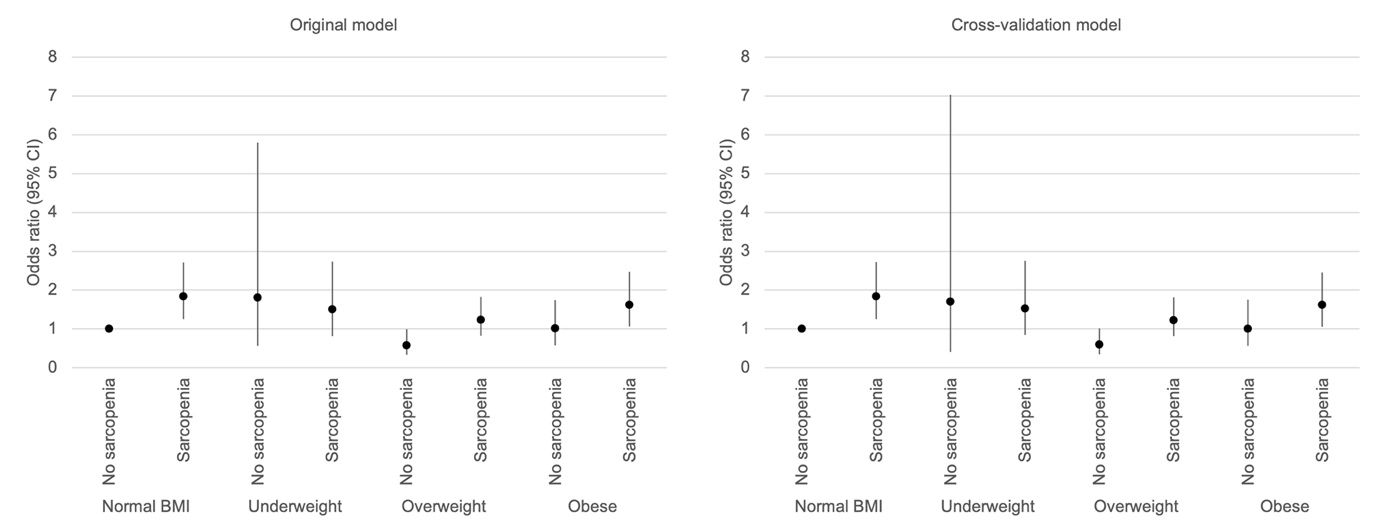


**Supplementary Figure 2.** Combined associations of body mass index and sarcopenia with mild cognitive impairment in the original model and the cross-validation model. Mild cognitive impairment was defined as a score of 12 or less out of 19 on the shorter version of the mini-mental state examination used in SABE Colombia, which is a valid screening tool. Sarcopenia was defined as low grip strength (<27 kg in men and <16 kg in women) or slow chair stands (>15 seconds in men and women). Ordinary logistic regression was used to create the original models (n=4,911). Cross-fit, partialing-out lasso logistic regression was used to create the cross-validation models (n=4,905). The reference group is normal BMI and no sarcopenia. The covariates in the original model and the controls in the cross-validation model were age, sex, height, education, income, civil status, smoking, and alcohol drinking. The Wald statistic for the exposure in the cross-validation model was 35.78 (p<0.001).

**Supplementary Table 1.** Combined associations of body mass index and sarcopenia with mild cognitive impairment, where sarcopenia is defined as low grip strength

|  | | Body mass index | | | |
| --- | --- | --- | --- | --- | --- |
|  | | Normal | Underweight | Overweight | Obesity |
| *Model 1* | |  |  |  |  |
|  | No sarcopenia | 1.00 (reference) n=914  (9% with MCI) | 1.16 (0.52, 2.57) n=64  (14% with MCI) | 0.77 (0.56, 1.07) n=1,210  (7% with MCI) | 0.86 (0.60, 1.24) n=781  (7% with MCI) |
|  | Sarcopenia | 2.14 (1.60, 2.86) n=796  (27% with MCI) | 1.92 (1.12, 3.30) n=96  (29% with MCI) | 1.24 (0.92, 1.69) n=781  (17% with MCI) | 1.76 (1.24, 2.51) n=380  (21% with MCI) |
| *Model 2* | |  |  |  |  |
|  | No sarcopenia | 1.00 (reference) n=900  (9% with MCI) | 1.10 (0.45, 2.66) n=59  (14% with MCI) | 0.76 (0.54, 1.07) n=1,184  (7% with MCI) | 0.94 (0.64, 1.39) n=772  (7% with MCI) |
|  | Sarcopenia | 2.01 (1.47, 2.75) n=771  (27% with MCI) | 1.60 (0.89, 2.87) n=94  (29% with MCI) | 1.26 (0.90, 1.75) n=759  (17% with MCI) | 1.92 (1.32, 2.82) n=372  (21% with MCI) |

Values are odds ratios (95% confidence intervals). Mild cognitive impairment (MCI) was defined as a score of 12 or less out of 19 on the shorter version of the mini-mental state examination used in SABE Colombia, which is a valid screening tool. Sarcopenia was defined as low grip strength (<27 kg in men and <16 kg in women). Model 1 was adjusted for age and sex (n=5,022). Model 2 was adjusted for age, sex, height, education, income, civil status, smoking, and alcohol drinking (n=4,911). N is number.

**Supplementary Table 2.** Combined associations of body mass index and sarcopenia with mild cognitive impairment, where sarcopenia is defined as slow chair stands

|  | | Body mass index | | | |
| --- | --- | --- | --- | --- | --- |
|  | | Normal | Underweight | Overweight | Obesity |
| *Model 1* | |  |  |  |  |
|  | No sarcopenia | 1.00 (reference) n=797  (11% with MCI) | 1.27 (0.54, 2.99) n=53  (15% with MCI) | 0.47 (0.32, 0.69) n=879  (5% with MCI) | 0.83 (0.55, 1.25)  n=497  (8% with MCI) |
|  | Sarcopenia | 1.47 (1.10, 1.96) n=913  (24% with MCI) | 1.34 (0.79, 2.28) n=107  (27% with MCI) | 1.02 (0.76, 1.37) n=1,112  (16% with MCI) | 1.11 (0.80, 1.55) n=664  (15% with MCI) |
| *Model 2* | |  |  |  |  |
|  | No sarcopenia | 1.00 (reference) n=779  (11% with MCI) | 1.05 (0.40, 2.73) n=49  (14% with MCI) | 0.55 (0.37, 0.82) n=864  (5% with MCI) | 0.96 (0.62, 1.49) n=495  (8% with MCI) |
|  | Sarcopenia | 1.57 (1.14, 2.14)  n=892  (23% with MCI) | 1.36 (0.76, 2.43) n=104  (27% with MCI) | 1.07 (0.78, 1.48) n=1,097  (15% with MCI) | 1.33 (0.93, 1.91) n=649  (15% with MCI) |

Values are odds ratios (95% confidence intervals). Mild cognitive impairment (MCI) was defined as a score of 12 or less out of 19 on the shorter version of the mini-mental state examination used in SABE Colombia, which is a valid screening tool. Sarcopenia was defined as slow chair stands (>15 seconds in men and women). Model 1 was adjusted for age and sex (n=5,022). Model 2 was adjusted for age, sex, height, education, income, civil status, smoking, and alcohol drinking (n=4,911). N is number.

**Supplementary Table 3.** Associations of body mass index with mild cognitive impairment in the original model and the cross-validation model

|  | Body mass index | | | |
| --- | --- | --- | --- | --- |
|  | Normal | Underweight | Overweight | Obesity |
| Original model | 1.00 (reference) | 1.61 (1.34, 1.95) | 0.76 (0.69, 0.84) | 0.74 (0.65, 0.83) |
| Cross-validation model | 1.00 (reference) | 1.62  (1.35, 1.95) | 0.76 (0.69, 0.84) | 0.74  (0.65, 0.83) |

Values are odds ratios (95% confidence intervals). Mild cognitive impairment was defined as a score of 12 or less out of 19 on the shorter version of the mini-mental state examination used in SABE Colombia, which is a valid screening tool. Ordinary logistic regression was used to create the original model (n=19,860). Cross-fit, partialing-out lasso logistic regression was used to create the cross-validation model (n=19,860). The covariates in the original model and the controls in the cross-validation model were age, sex, height, education, income, civil status, smoking, and alcohol drinking. The Wald statistic for body mass index in the cross-validation model was 84.65 (p<0.001).

**Supplementary Table 4.** Associations of grip strength and chair stands with mild cognitive impairment in the original models and the cross-validation models

|  | | Normal | Low or slow |
| --- | --- | --- | --- |
| *Grip strength* | |  |  |
|  | Original model | 1.00 (reference) | 1.90 (1.56, 2.31) |
|  | Cross-validation model | 1.00 (reference) | 1.90 (1.56, 2.32) |
| *Chair stands* | |  |  |
|  | Original model | 1.00 (reference) | 1.61 (1.31, 1.98) |
|  | Cross-validation model | 1.00 (reference) | 1.61 (1.31, 1.98) |

Values are odds ratios (95% confidence intervals). Mild cognitive impairment was defined as a score of 12 or less out of 19 on the shorter version of the mini-mental state examination used in SABE Colombia, which is a valid screening tool. The cut-off for low grip strength was <27 kg in men and <16 kg in women. The cut-off for slow chair stands was >15 seconds in men and women. Ordinary logistic regression was used to create the original models (n=5,000 for grip strength and chair stands). Cross-fit, partialing-out lasso logistic regression was used to create the cross-validation models (n=4,998 for grip strength and n=4,990 for chair stands). The covariates in the original model and the controls in the cross-validation model were age, sex, height, education, income, civil status, smoking, and alcohol drinking. The Wald statistic for grip strength in the cross-validation model was 40.32 (p<0.001). The Wald statistic for chair stands in the cross-validation model was 20.11 (p<0.001).
